# Bilaterales Karpaltunnelsyndrom bei einem Patienten mit malignem Melanom unter adjuvanter Pembrolizumab-Therapie

**DOI:** 10.1007/s00105-025-05480-6

**Published:** 2025-05-02

**Authors:** S. Weyer-Fahlbusch, M. Sandersfeld, C. Ritthaler, L. Hauck, P. Dücker, L. Susok, T. Gambichler

**Affiliations:** 1 Klinik für Dermatologie, Klinikum Dortmund, Universität Witten/Herdecke, Dortmund, Deutschland; 2 Klinik für Dermatologie, Christliches Klinikum Unna, Unna, Deutschland; 3 Klinik für Neurologie, Klinikum Dortmund, Universität Witten/Herdecke, Dortmund, Deutschland; 4 Klinik für Dermatologie, Ruhr-Universität Bochum, Bochum, Deutschland

**Keywords:** Neurologische Nebenwirkungen, Karpaltunnelsyndrom, Immuncheckpointinhibitor, Autoimmunvermittelte Nebenwirkungen, Melanom, Neurological side effects, Carpal tunnel syndrome, Immune checkpoint inhibitor, Autoimmune-mediated side effects, Melanoma

## Abstract

Immuncheckpointinhibitoren (ICI) werden bei multiplen malignen Erkrankungen erfolgreich eingesetzt. Zum Nebenwirkungsspektrum gehören neben dermatologischen, endokrinologischen, gastrointestinalen und hepatischen auch selten neurologische Nebenwirkungen des zentralen und häufiger des peripheren Nervensystems. Vorbestehende neurologische Erkrankungen können sich verschlechtern. Das bilateral auftretende Karpaltunnelsyndrom (KTS) tritt selten auf. Im vorliegenden Fall kam es bei einem 83-jährigen Patienten mit malignem Melanom (MM) im Stadium IIIC nach 5 adjuvanten Therapiezyklen mit Pembrolizumab 200 mg zu Schmerzen, Schwellungen und Parästhesien in beiden Händen. Nach Diagnostik eines beidseitigen KTS wurde eine Prednisolon-Stoßtherapie durchgeführt und auf eine Erhaltungsdosis von 20 mg p.o. reduziert. Trotz notwendiger Eskalation der ICI-Therapie auf Ipilimumab/Nivolumab bei Progress des MM konnte unter begleitender Prednisolon-Therapie und Physiotherapie das KTS verbessert werden.

## Abstract (en)

Immune checkpoint inhibitors (ICIs) are successfully used in multiple malignant diseases. Their spectrum of adverse effects includes dermatological, endocrinological, gastrointestinal, and hepatic toxicities, as well as rare neurological side effects affecting the central nervous system and more frequently the peripheral nervous system. Pre-existing neurological disorders may deteriorate. Bilateral carpal tunnel syndrome (CTS) rarely occurs. In the present case, an 83-year-old patient with stage IIIC malignant melanoma (MM) developed pain, swelling, and paresthesia in both hands after five adjuvant therapy cycles with pembrolizumab 200 mg. Following the diagnosis of bilateral CTS, pulse therapy with prednisolone was initiated and subsequently reduced to a maintenance dose of 20 mg orally. Despite the necessary escalation of ICI therapy to ipilimumab/nivolumab due to MM progression, CTS improved under concomitant prednisolone therapy and physiotherapy.

## Fallbericht

### Patient

Ein 83-jähriger Patient mit der Diagnose eines malignen Melanoms im Stadium IIIC (pT4b, N2c, M0; ED 07/2023) stellte sich 01/2024 mit progredienter ödematöser Schwellung der Hände vor. Diese waren von Parästhesien der Digiti 1–3 beidseits begleitet. Der Patient befand sich bereits seit 09/2023 unter einer adjuvanten Therapie mit Pembrolizumab. Die oben genannte Symptomatik begann eine Woche nach der zweiten Pembrolizumab-Gabe und stellte sich im Verlauf progredient dar; zuvor habe er nie unter ähnlichen Beschwerden gelitten. Bei dem Patienten sind neben einem Diabetes mellitus Typ 2 keine weiteren relevanten Erkrankungen bekannt.

### Klinischer Befund

**Abb. 1:**
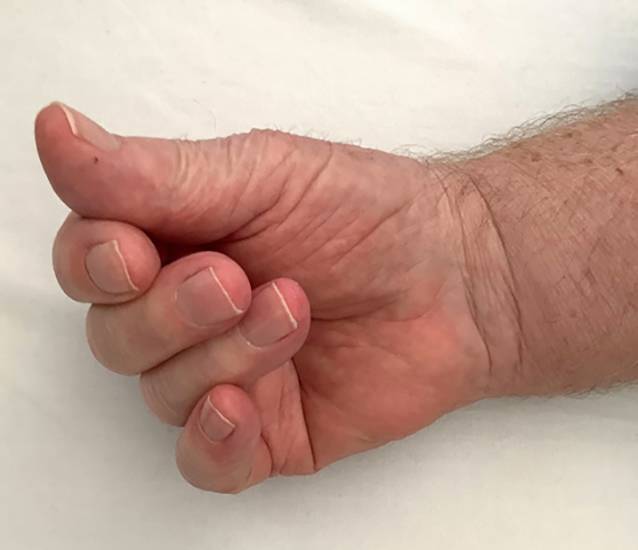
Unvollständiger Faustschluss des Patienten mit Karpaltunnelsyndrom unter Therapie mit Pembrolizumab

Bei der Inspektion zeigte sich eine ödematöse Schwellung der Hände beidseits mit Schmerzen (NRS [numerische Rating-Skala] 8/10) sowie Parästhesien im Bereich der Digiti 1–3 beidseits. Der Faustschluss war beidseitig, insbesondere rechts, nur eingeschränkt möglich **(**Abb. 1**)**.

### Weiterführende Diagnostik

**Abb. 2:**
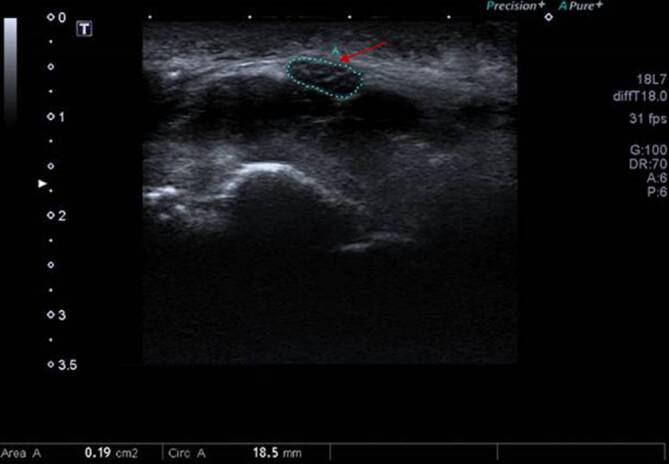
Nervensonographie 02/2024: Es zeigt sich ein klassischer Befund eines Karpaltunnelsyndroms mit Schwellung des N. medianus (siehe *roter Pfeil* und *grün gepunktetes Oval*) vor dem Eintritt in den Karpaltunnel

**Abb. 3:**
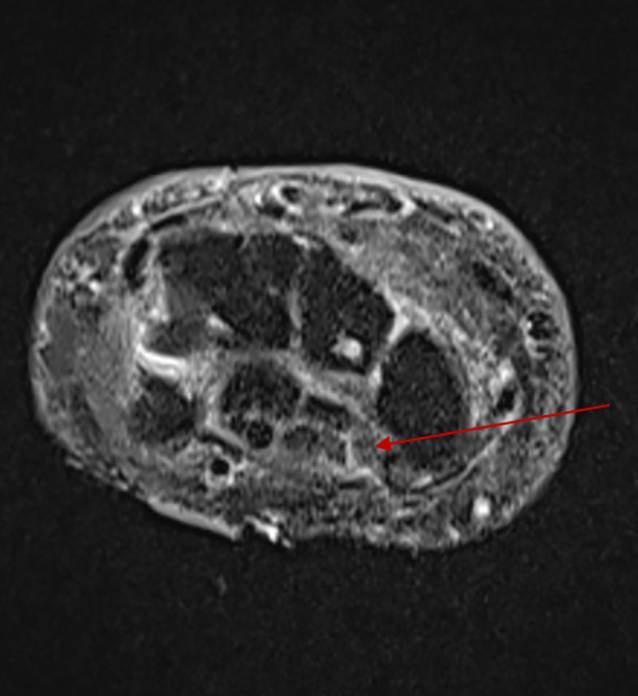
Magnetresonanztomographie des linken Handgelenkes nativ und nach KM (Kontrastmittel) 02/2024: Es zeigt sich das Bild eines Karpaltunnelsyndroms bei abgeflachtem N. medianus mit geringem Ödem im Karpaltunnel (*roter Pfeil*). Diffuses subkutanes Ödem. Kein Nachweis einer Tendosynovitis

Laborchemisch zeigten sich bis auf eine Hypertriglyzeridämie von 347 mg/dl (Referenz < 150) und eine Reduktion des HDL(High Density Lipoprotein)-Cholesterins auf 34 mg/dl (Referenz > 40) keine Auffälligkeiten. In der konsiliarischen neurologischen Diagnostik stellte sich im Ultraschall eine Schwellung des N. medianus beidseits dar **(**Abb. 2**)**. In der Elektroneurographie zeigten sich eine verlängerte distale motorische Latenz sowie reduzierte Muskelsummenaktionspotenziale. Des Weiteren wurde ein Ausfall der sensiblen Nervenaktionspotenziale festgestellt (rechts > links). In der Magnetresonanztomographie des rechten Handgelenks ergab sich das Bild eines Karpaltunnelsyndroms (KTS) bei abgeflachtem N. medianus mit Ödem im Karpaltunnel und diffusem, subkutanem Ödem ohne Nachweis einer Tendosynovitis **(**Abb. 3**)**.

### Diagnose und Verlauf

In Zusammenschau der Befunde und Vorgeschichte wurde ein ICI(Immuncheckpointinhibitoren)-induziertes bilaterales KTS diagnostiziert. Es erfolgte die Einleitung einer intravenösen Prednisolon-Stoßtherapie mit initial 250 mg Solu-Decortin H (SDH) unter engmaschigen Kontrollen der Blutglukosewerte. Unter dieser Therapie zeigten sich die Symptome bereits nach zwei Tagen rückläufig. Bei neu aufgetretenen subkutanen Metastasen im Bereich der Narbe der vorherigen Lymphknotenexzision axillär rechts wurde eine Eskalation der Systemtherapie auf Ipilimumab/Nivolumab durchgeführt. Eine Reduktion der Prednisolon-Therapie auf eine Erhaltungsdosis von 20 mg per os unter gleichzeitiger Gabe von Protonenpumpeninhibitoren und Vitamin-D-Substitution konnte erfolgen. Unterstützend wurden zur Ruhigstellung und Entlastung Unterarmschienen in der Nacht getragen und Physiotherapie durchgeführt. Hierunter zeigte sich der Befund weiterhin stabil, sodass bisher keine operative Intervention notwendig war.

## Diskussion

Das KTS ist mit einer Inzidenz von 3,5 auf 1000/Jahr das häufigste Nervenkompressionssyndrom. Häufige Risikofaktoren sind genetische Prädisposition, eine hohe Belastung der Handgelenke, Adipositas und endokrinologische Erkrankungen wie der Diabetes mellitus. Außerdem ist das KTS eine bekannte Nebenwirkung von Aromataseinhibitoren der 3. Generation. Hier zeigte sich oft 6 bis 8 Wochen nach der Initiierung der Therapie eine Karpaltunnelsymptomatik, woraufhin fast 70 % der Patienten vorzeitig die Therapie abbrechen mussten und onkologische Therapieprotokolle nicht wie vorgesehen durchgeführt werden konnten. Bisphosphonate und Fluorchinolone stellen ebenfalls ein erhöhtes Risiko für das Auftreten eines KTS dar. Männer sind deutlich seltener betroffen als Frauen (Verhältnis von 1:3,5). Der Erkrankungsgipfel liegt zwischen dem 40. und 70. Lebensjahr [1, 12, 18]. Glukokortikoide in Verbindung mit Physiotherapie haben in der Regel einen guten therapeutischen Effekt. Bei schweren, therapieresistenten oder rezidivierenden Fällen kommen – wie beim klassischen KTS – auch operative Maßnahmen in Betracht.

Immuncheckpointinhibitoren (z. B. Nivolumab, Pembrolizumab, Ipilimumab, Avelumab) stellen eine relativ neue und gezielte immunologische Therapiemöglichkeit dar, die bei diversen malignen Tumoren (z. B. Melanom, nichtkleinzelliges Lungenkarzinom, Nierenzellkarzinome, HNO[Hals-Nasen-Ohren]-Tumoren) zunehmend zum Einsatz kommt. Neben den bekannten dermatologischen, endokrinologischen, gastrointestinalen und hepatischen Nebenwirkungen können auch – allerdings in weitaus geringerem Ausmaß – immunvermittelte neurologische Nebenwirkungen auftreten. Zu den immunmediierten Komplikationen im zentralen Nervensystem zählen Enzephalitiden wie auch die Hirnstammenzephalitis. Unter Therapie mit Nivolumab wurden auch Fälle mit zerebralen Ischämien beschrieben. Ebenso existieren Fallberichte über das neue Auftreten von demyelinisierenden zentralnervösen Erkrankungen (z. B. Optikusneuritis, transverse Myelitis) innerhalb von Wochen bis Monaten nach ICI-Therapie wie auch sehr seltene Manifestationen des zentralen Nervensystems (ZNS) (z. B. Tolosa-Hunt-Syndrom, aseptische Meningitis, granulomatöse ZNS-Inflammation). Bereits vorbestehende neurologische Erkrankungen (z. B. multiple Sklerose) können sich unter ICI-Therapie verschlechtern [4, 7, 8, 13, 17, 20]. Das periphere Nervensystem ist allerdings deutlich häufiger von ICI-mediierten Nebenwirkungen betroffen als das zentrale Nervensystem. Die bisweilen am häufigsten berichtete neuromuskuläre Komplikation stellt die ICI-vermittelte Myasthenia gravis dar, die v. a. unter PD-1-Inhibitoren aufzutreten scheint. Dazu kann es zusätzlich auch zu Polymyositiden kommen, teilweise mit Begleitmyokarditis und okulobulbärer Symptomatik. Akute und chronische Formen von demyelinisierenden Neuropathien mit motorischer oder sensorischer Beteiligung scheinen ebenso zum Nebenwirkungsspektrum der ICI zu gehören. Zu den schwerwiegenden Formen zählt das ICI-assoziierte Guillain-Barré-Syndrom. Ebenso wurden chronisch inflammatorische demyelinisierende Polyneuropathien, vaskulitische Neuropathien, brachiale Plexusneuritiden und Fazialisparesen unter ICI-Therapie berichtet [3, 7, 8, 17, 20]. Das Karpaltunnelsyndrom (KTS) – ein Engpasssyndrom mit Druckschädigung des N. medianus bei seinem Durchtritt durch den Karpaltunnel am Handgelenk – wurde bisher nur sehr selten in der Literatur als Nebenwirkung einer ICI-Therapie berichtet [2, 5, 6, 11, 15, 16, 19].

Im vorliegenden Fall hatte der Patient zwar einen lange bekannten Diabetes mellitus, aber der zeitliche Zusammenhang zur ICI-Einleitung und das beidseitige Befallsmuster sprechen eher für ein ICI-induziertes KTS. In Tab. 1 sind die bisher in internationalen Zeitschriften publizierten Fälle mit ICI-induziertem KTS aufgelistet [2, 5, 6, 11, 15, 16, 19]. Das mediane Manifestationsalter mit 74 Jahren (Spannweite: 40–97) liegt etwas höher als beim klassischen KTS [1, 12, 18]. Die beim klassischen KTS zu beobachtende Gynäkotropie ist beim ICI-induzierten KTS mit einem Geschlechtsverhältnis von 1:1 bisher nicht festzustellen, wenngleich die bisher berichtete Population mit ICI-induziertem KTS klein ist. Das ICI-induzierte KTS war nur bei 2 von 21 Patienten unilateral beschrieben worden, allerdings tritt das klassische KTS auch überwiegend bilateral auf [1, 12, 18]. Das ICI-assoziierte KTS tritt im Median nach 3 Monaten nach Therapieeinleitung auf, wobei überwiegend Anti-PD-1-Inhibitoren eingesetzt wurden. Leider wurde nur selten berichtet, ob die ICI beendet oder wieder eingeleitet wurde. Bei über der Hälfte der Patienten wurden die ICI im adjuvanten Setting eingesetzt, was sich mit der allgemein höheren Patientenanzahl im adjuvanten Setting mit ICI-Indikation deckt. Die häufigsten Behandlungsformen bestanden aus systemischen Prednisolon-Gaben und/oder Kortisoninfiltrationen, die in den meisten Fällen eine Besserung der Symptomatik bewirkten.

**Tab. 1:** Literaturübersicht der Patientenfälle mit Karpaltunnelsyndrom (KTS) unter ICI(Immuncheckpointinhibitoren)-Therapie

Patient [Referenz]	Alter/Geschlecht	Nebendiagnosen	Tumortyp	Onkologische Therapie	Therapieform	Therapiezeit bis KTS (Monate)	KTS Lateralität	Therapie des KTS
1 [19]	79/m	Zustand nach Myokardinfarkt	Plattenepithelkarzinom der Lunge	Carboplatin, Atezolizumab, + nab-Paclitaxel, (2 Zyklen), Lungenteilresektion, 4 Zyklen Monotherapie, Atezolizumab	Neoadjuvant	1,5	Bilateral, re > li	Operation rechtsseitig: persistierende Symptomatik, Prednisolon (systemisch): keine Wirkung, IVIG über 4 Monate mit Erfolg
2 [19]	70/w	KA	Nierenzellkarzinom	Pazopanib: Progress, Nivolumab 3 mg/kgKG	Palliativ	18	Bilateral, re > li	Prednisolon (systemisch), im Verlauf ICI Re-Challenge unter einer Erhaltungsdosis von 10 mg Prednisolon
3 [19]	71/m	KA	Malignes Melanom, BRAF-wt	Nivolumab (12 Zyklen, alle 2 Wochen), dann erhöhte Dosis 480 mg alle 4 Wochen	Palliativ	8	Bilateral	Prednisolon (systemisch): klinische Besserung, Erhaltungstherapie: Pregabalin/NSAIDs
4 [19]	68/m	KA	Malignes Melanom, BRAF-wt	Nivolumab 1 mg/kgKG, Ipilimumab 3 mg/kgKG (4 Zyklen): partielles Ansprechen, Nivolumab-Monotherapie, dann zusätzlich MTX	Palliativ	12	Bilateral, re > li	Prednisolon (systemisch): Exanthem, persistierende Beschwerden, nach Hinzunahme von MTX 15 mg/Woche Besserung der Beschwerden
5 [5]	77/w	Arterielle Hypertonie, Osteoarthritis	Malignes Melanom, BRAF-wt	Pembrolizumab	Adjuvant	16	Bilateral	ICI abgesetzt Kortisoninfiltration, Pregabalin, Prednisolon (systemisch), Naproxen, Gabapentin: ohne Erfolg, Operation (erfolgreich)
6 [5]	66/w	Diabetes mellitus, Gicht, arterielle Hypertonie	Malignes Melanom, BRAF-wt	Pembrolizumab	Adjuvant	5	Bilateral	ICI fortgeführt, Operation links (erfolgreich), Kortisoninfiltration rechts (mäßig erfolgreich)
7 [5]	57/m	KA	Malignes Melanom, BRAF-mut	Vemurafenib und Dabrafenib, Pembrolizumab	Adjuvant	3	Bilateral	ICI abgesetzt zur vollständigen Remission, Kortisoninfiltration, Wiederauftreten mit erneuter Infiltration
8 [5]	74/w	Mammakarzinom	Malignes Melanom, BRAF-mut	Dabrafenib und Trametinib, Pembrolizumab	Adjuvant	3	Bilateral	ICI fortgeführt, Stützschienen zur Nacht: Besserung der Symptomatik
9 [5]	40/m	Alkohol- und Nikotinabusus	Malignes Melanom, BRAF-wt	Ipilimumab/Nivolumab	Palliativ	2	Bilateral	ICI abgesetzt wegen Progress, Pregabalin, Amitriptylin, Kortisoninfiltration: ohne Besserung, Prednisolon (systemisch): klinische Besserung
10 [5]	86/m	Follikuläres B‑Zell-Lymphom in kompletter Remission	Malignes Melanom, BRAF-mut	Nivolumab	Adjuvant	3	Bilateral	ICI abgesetzt wegen Progress, Prednisolon (systemisch): klinische Besserung
11 [5]	83/m	Zustand nach malignem Melanom Stadium I, Vorhofflimmern, Epilepsie	Multiple kutane Plattenepithelkarzinome	Pembrolizumab	Adjuvant	16	Unilateral	ICI abgesetzt wegen Grad-III-Hepatitis, Kortisoninfiltration: keine klinische Besserung
12 [5]	72/w	Arterielle, Hypertonie, Diabetes mellitus	Malignes Melanom, BRAF-mut	Ipilimumab 4 Zyklen, Dabrafenib und Trametinib, Vemurafenib und Cobimetinib, Pembrolizumab	Adjuvant	24	Bilateral	ICI abgesetzt bei „stable disease“, Prednisolon (systemisch): keine klinische Besserung, Operation: klinische Besserung
13 [5]	78/w	Arterielle Hypertonie, Osteoarthritis, Zustand nach KTS rechts mit Operation 1955	Malignes Melanom, BRAF-wt	Nivolumab	Adjuvant	1,5	Unilateral li	ICI fortgeführt, Prednisolon (systemisch): mäßige Besserung, Operation: deutliche Besserung
14 [5]	86/w	Zustand nach Lungenarterienembolie, Schrittmacherimplantation bei atrioventrikulärem Block, arterielle Hypertonie	Malignes Melanom, BRAF-wt	Nivolumab	Adjuvant	3	Bilateral	ICI fortgeführt, Prednisolon (systemisch): klinische Besserung
15 [5]	88/w	Diabetes mellitus, arterielle Hypertonie	Merkel-Zell-Karzinom	Carboplatin und Etoposid, Avelumab	Adjuvant	12	Bilateral	ICI fortgeführt, Kortisoninfiltration: ohne deutliche Besserung
16 [2]	53/m	KA	Nierenzellkarzinom	Neoadjuvant Axitinib, radikale Nephrektomie links, adjuvant Pembrolizumab	Adjuvant	3	Bilateral	ICI abgesetzt, Prednisolon (systemisch): klinische Besserung
17 [2]	56/w	KA	Malignes Melanom, BRAF-wt	Nivolumab	Adjuvant	1	Bilateral	Prednisolon (systemisch): klinische Besserung, Re-Challenge: erneute Symptomatik, ICI abgesetzt, erneut Prednisolon systemisch: klinische Besserung
18 [16]	97/m	KA	Urothelkarzinom	Pembrolizumab	Palliativ	1	Bilateral	ICI abgesetzt, keine KTS-Therapie, nach 3 Wochen Hospiz
19 [16]	81/m	KA	KA	Pembrolizumab	KA	1,5	Bilateral	ICI abgesetzt, keine KTS-Therapie
20 [6]	47/w	Gleichzeitiges Auftreten einer systemischen Sklerose	Malignes Melanom, BRAF-wt	Nivolumab	Palliativ	2	Bilateral	ICI abgesetzt, niedrig dosierte Kortikosteroide per os und Kortisoninjektionen, Befundbesserung
21 – vorliegender Fall [9]	83/m	Diabetes mellitus	Malignes Melanom, BRAF-wt	Pembrolizumab 7 Gaben, Umstellung auf Ipilimumab/Nivolumab, 3 Zyklen, „stable disease“	Adjuvant	2,2	Bilateral re > li	ICI nicht abgesetzt, Prednisolon (systemisch), rückläufige Symptomatik ab Tag 2

*Re* rechts, *li* links, *IVIG* intravenöse Immunglobulinersatztherapie, *KA* keine Angaben, *KG* Körpergewicht, *NSAIDs* nichtsteroidale Antirheumatika, *MTX* Methotrexat

Differentialdiagnostisch kommt als ICI-Komplikation neben der rheumatoiden Polyarthritis insbesondere die sich ähnlich darstellende remittierende seronegative symmetrische Synovitis mit eindrückbarem Ödem (RS3PE) in Betracht, die ebenso mehrfach in Verbindung mit malignen Tumoren (paraneoplastisch), aber auch mit ICI-Therapie beschrieben worden ist [10, 11, 14]. Im Gegensatz zum klassischen KTS tritt das RS3PE-Syndrom meist bei älteren Männern auf (4:1). Es ist durch rasch auftretende symmetrische Ödeme der Handrücken sowie Beschwerden der proximalen Metakarpophalangeal- und Interphalangealgelenke der Hände gekennzeichnet. Die Ödeme sind nicht erythematös, kalt, weich und eindrückbar. Häufig besteht aufgrund der Schmerzen eine sehr starke Funktionseinschränkung. Der Rheumafaktor und die antinukleären Antikörper (ANAs) sind stets negativ. Sonographisch zeigt sich im Vergleich zum KTS eine Flüssigkeitsanreicherung im Bereich der Sehnenscheide (Tendinitis). Interessanterweise kann das RS3PE-Syndrom auch mit dem KTS assoziiert auftreten, wenn das Ödem im Karpaltunnel durch internen Druck ein zu großes Ausmaß annimmt [1, 10, 12, 14].

Gemäß aktueller Leitlinie für die Behandlung des klassischen KTS werden im Frühstadium der Erkrankung, wenn lediglich Reizsymptome wie z. B. nächtliche Parästhesien bestehen, konservative Behandlungsversuche empfohlen. Hierzu gehören z. B. Handgelenkschienen über Nacht, systemische Glukokortikoide oder ultraschallgesteuerte intraläsionale Glukokortikoide. Bei anhaltenden sensiblen und/oder motorischen Ausfallserscheinungen, wie z. B. schmerzhaften Parästhesien, soll eine Operation erfolgen. Die zugrunde liegende Pathophysiologie des ICI-induzierten KTS ist unklar. Eine Dysregulation peripherer T‑Zellen und/oder auch bereits zuvor subklinisch bestehende Entzündungen, die durch die ICI hochreguliert werden, könnten beim ICI-induzierten KTS eine Rolle spielen [2, 5, 6, 11, 15, 16, 19]. Daher scheinen zunächst antiinflammatorische Therapieansätze mit Glukokortikoiden beim ICI-induzierten KTS gerechtfertigt zu sein. Zusammenfassend kann festgestellt werden, dass das KTS – insbesondere bei bilateraler Symptomatik – als Nebenwirkung im Rahmen einer ICI-Therapie bedacht werden sollte.
